# A Two-Dimensional Laser Scanning Mirror Using Motion-Decoupling Electromagnetic Actuators

**DOI:** 10.3390/s130404146

**Published:** 2013-03-27

**Authors:** Bu Hyun Shin, Oh Dongho, Seung-Yop Lee

**Affiliations:** 1 Department of Mechanical Engineering, Sogang University, 1 Shinsu-dong, Mapo-gu,Seoul 121-742, Korea; 2 Department of Mechanical Engineering, Chungnam National University, 99 Deahangno, Yuseong-gu, Daejeon 305-764, Korea

**Keywords:** laser scanning mirror, electromagnetic actuator, optical scanning, two-dimensional scanner

## Abstract

This work proposes a two-dimensional (2-D) laser scanning mirror with a novel actuating structure composed of one magnet and two coils. The mirror-actuating device generates decoupled scanning motions about two orthogonal axes by combining two electromagnetic actuators of the conventional moving-coil and the moving-magnet types. We implement a finite element analysis to calculate magnetic flux in the electromagnetic system and experiments using a prototype with the overall size of 22 mm (W) × 20 mm (D) × 15 mm (H) for the mirror size of 8 mm × 8 mm. The upper moving-coil type actuator to rotate only the mirror part has the optical reflection angle of 15.7° at 10 Hz, 90°at the resonance frequency of 60 Hz at ±3V(±70mA) and the bandwidth of 91 Hz. The lowermoving-magnet type actuator has the optical reflection angle of 16.20°at 10 Hz and50°at the resonance frequency of 60 Hz at ±5V(±34mA) and the bandwidth of 88 Hz. The proposed compact and simple 2-D scanning mirror has advantages of large 2-D angular deflections, wide frequency bandwidth and low manufacturing cost.

## 1. Introduction

2-D scanning mirror systems have been widely used in commercial and industrial fields such as barcode reading systems, display projectors, optical sensors and display devices. In a two-dimensional (2-D) scanning mirror system, an optical mirror is rotated about two orthogonal axes to reflect a light beam on a target plane. Various 2-D scanning mirror systems using micro-electro-mechanical systems (MEMS)have been reported. The MEMS based 2-D actuators have high dynamic performance at the resonance frequency, but their low dynamic ranges are their weak points for some applications. Electrostatic actuation is widely used for MEMS based optical devices [1–4], but the main drawback is relatively high voltages over 40V required for the large angular motion of the scanning mirror.

Some 2-D scanning mirrors were also introduced using the thermally actuated method [5,6], piezoelectric materials [7–11] andelectromagnetically actuators [12–18]. Electromagnetic actuation uses a lower voltage to generate relatively large forces, enabling the scanning mirror to have a large deflection angle and reliable operation. TheMEMS based, electromagnetically actuated 2-D scanning mirrors with moving-coil types [12,13] or with steel materials [14] have large reflection angles at the resonance frequency. On the other hand, the MEMS based electromagnetic actuators with moving-magnet types have a broad dynamic range and static pointing performance [15,16]. Other types of electromagnetic actuators without MEMS process use soft magnetic films [17] and a singleiron-bead [18] to generate 2-D motions using one solenoid at lower resonance frequency than those of other MEMS based mirror systems.

Some electromagnetic actuators have been introduced in the 2-D scanning mirror systems with large angular deflections and high dynamic performances [19,20]. However, their complex structures and bulky sizes become drawbacks for the 2-D actuation in many applications such as confocal laser scanning microscopes, optical coherence topographies, and scanning laser rangefinders, projectors and laser marking machines.

In this paper, we present a miniaturized scanning mirror system using electromagnetic actuators with a simpledriving structure for the decoupled 2-D motions. The new scanning mirror system generates the 2-D swing motions of the mirror by combining two electromagnetic actuators of the conventional moving-coil and the moving-magnet types. Experiments using a prototype show large angular deflection at low frequencies, moderate bandwidth, static performance and low manufacturing cost.

## 2. Design of a Scanning Mirror

**Figure 1.**
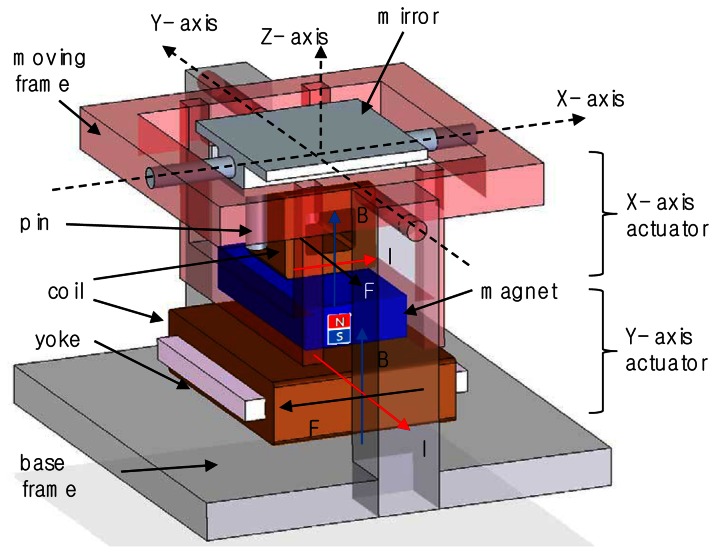
Design and mechanism of the proposed 2-D scanning mirror actuator: Two actuators generate the mirror-rotating force (F) around X-axis and the frame-rotating force around Y-axis based on the magnetic flux (B) and currents (I).

In 2-D scanning mirror systems, the beam from laser or light source is sent to the mirror and the beam reflected from the mirror is focused onto the target surface. As the position of the mirror is deflected by 2-D rotary actuators, the focused beam creates a 2-D beam pattern on the target image plane. The basic concept of the proposed mirror-actuating structure is shown in Figure 1. The new 2-D scanning mirror system has a novel structure to combine two electromagnetic actuators of the moving coil and the moving magnet types for the decoupled 2-D motions. The scanning mirror systemis based on a 2-D gimbal structure with rotational joints for independent 2-D motions. The two actuators generate two orthogonal motions: the rotation of the mirror part around X-axis and the rotation of the whole frame around Y-axis. In order to rotate only the mirror part about the X-axis, the coilis wound around the Y-axis in a clockwise directionand it is attached to the backside of the mirror located within the moving frame, acting as the moving-coil type.The direction of the magnetic flux is onZ-axis and electrical current flows along X-axis. Therefore the electromagnetic force is generated along Y-axis to rotate the mirror around X-axis based on Lorentz's law. In contrast, in order to swing the whole frame around Y-axis, the magnet is attached on the moving frame over the coillocated on the base frame, acting as the moving-magnet type. The coil is wound around the X-axis in a counterclockwise direction on the base frame. The second actuator generates the electromagnetic force along the X-axis to rotate the whole frame around Y-axis since the magnetic flux is in the Z direction and the current flows along Y-axis.Here the electromagnetic force induced by coils close to magnet is dominant compared to one induced by coils far from magnet.

## 3. Dynamic Model

Two types of driving forces are generated in each actuator. Since the retract pin is attached to the coil of the mirror, the magnetic force between the retract pin and the magnet holds the mirror at the central position. Similarly, the magnetic force between the magnet and the core yoke holds the moving frame at the central position. The electromagnetic force generates the rotational motion on each axis when the current is applied to each coil. The rotational direction of is controlled by the direction of the current applied to each coil. The dynamic model of the electromagnetic system is based on DC motors. Therefore, the equation of the dynamic system is written by:

(1) $$J\ddot{\theta }+F_{\text{magnetic}}=F_{\text{elecromagnetic}}$$

where *J* is the mass moment of inertia and *θ̈* is the angular acceleration. Although the magnetic and electromagnetic forces *F_magnetic_* and *F_electromagnetic_* are nonlinear, the dynamic model of the 2-D scanning mirror system is linearizedin order to approximatethe dynamic performances of the system such asthe resonance frequency, bandwidth and rotational angle. Then the magnetic and electromagnetic forces can be written as:

(2) $$F_{\text{magnetic}}=K_{m}\;\theta$$

(3) $$F_{\text{elecromagnetic}}=K_{t}\;I$$

where *θ* is the rotation angle. *K_m_* and *K_t_* are the restoring (spring) constant and the torque constant, respectively. *I* is the current flowing through the coil. If the inductor component is ignored in the electric circuit, the circuit equation becomes:

(4) $$V=RI+Ke\;\dot{\theta }$$

Here *V* is the applied voltage and *R* is the resistance of coil. And *θ̇* is the angular velocity and *K_e_* is the back-emf constant. In general, the back-emf constant has the same value as the torque constant in the case of DC motors. The back-emf constant multiplied by the angular velocity makes the back-emf voltage. Based on the linear Equation (4), the coil current is calculated using the input voltage and angular velocity at the moment. In the static operation, the current becomes constant, which is V/R, since the angular velocity vanishes. However, in dynamic states including resonance frequency, the sign and magnitude of the back-emf voltage change due to the varied angular velocity. Since the proposed 2-D mirror system oscillates like a pendulum, the back-emf voltage could be positive or negative. The maximum current of coil is less or larger than V/R due to the sign of the back-emf voltage. When the back-emf voltage is negative, the current of coil is larger than V/R. The additional current of coil induced by the back-emf voltage is the regenerative (circulating) one since the DC motor system acts as a generator. However, the power supply of the system has the maximum current of V/R at the given input voltage, when the direction of the angular velocity changes.

By combining the Equations (1) and (4), we obtain the second-order linear equation. Using Laplace transform, the ratio of the input voltage to the output angle becomes:

(5) $$\frac{\Theta }{V}=\frac{1}{\frac{JR}{K_{t}}s^{2}+K_{e}\;s+\frac{K_{m}R}{K_{t}}}$$

**Figure 2.**
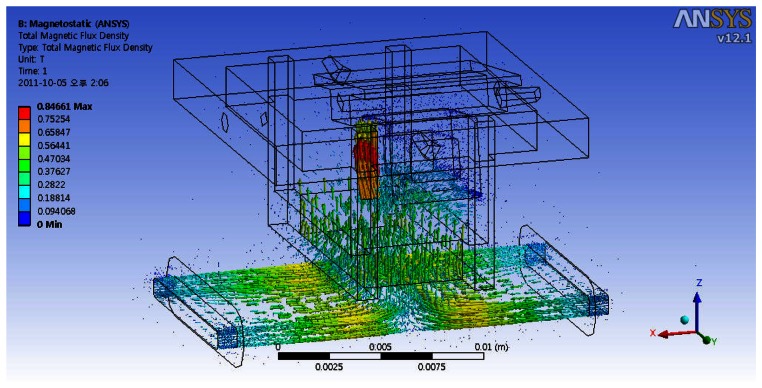
Simulation results of magnetic flux for the rotation by 10 degrees on the X-axis.

We implement a finite element analysis (FEA) to calculate the magnetic and electromagnetic forces in the electromagnetic system using a commercial program ANSYS. Figure 2 shows the simulation results on magnetic flux density for the rotational motion of 10 degrees in the X direction. The direction of magnetic flux is the same to that of the design model shown in Figure 1. The magnitude of magnetic flux density is used to calculate the magnetic and electromagnetic forces. Then, the simulated parameters of the restoring and torque constants are obtained from the simulation results of magnetic and electromagnetic forces using Equations (2) and (3).

## 4. Experiments

### 4.1. Experimental Setup

**Figure 3.**
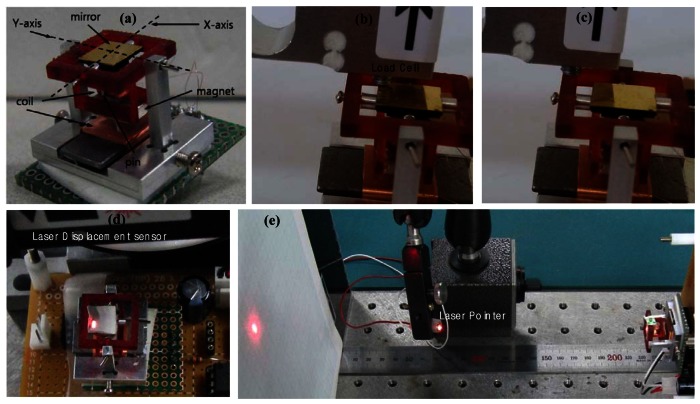
The experiment setup to measure reflection angles. (**a**) A prototype of the 2-D mirror scanning system, (**b**) the measurement of the torque of the mirror by a load cell, (**c**) the measurement of the torque of the frame by a load cell, (**d**) the displacement measurement of mirror, (**e**) the measurement of laser beam.

In order to measure the dynamic performances of the 2-D scanning mirror system, we manufactured a prototype as shown in Figure 3(a). The thin glass mirror is attached to the plastic mirror mold. And then the coil and pin are inserted into the plastic mirror mold. The size of the mirror is 8 mm × 8 mm and the overall size of the scanning mirror system is 22 mm (W) × 20 mm (D) × 15 mm (H). The moving frame is made of plastic using a rapid prototyping process to reduce inertia. The total mass of the moving parts is 1.45g. Magnet material is ND45. The coil in the mirror part has 712 turns and 42 Ω, but the coil at the base has 1,312 turns and 145Ω. The material of the core yoke of the base coil is steel (S45C) and the material of the base frame is duralumin (A7075).

We implement experiments using the prototype. The open-loop control circuit using power op-amps is used and the input signals come from a function generator. A load cell (CAS^tm^ PW4M C3) is used to measure the torques of the mirror and whole frame in order to calculate the restoring and torque constants, as shown in Figure 3(b,c). The laser displacement sensor (Keyence^tm^ LB 081) is also used to measure the displacement of the mirror as shown in Figure 3(d).The vertical motion of the mirror is measured at the corner of the mirror which is 3mm × 3mm away from the center of the mirror. Then the rotational angle is calculated using trigonometric functions.

The optical reflection angle of the mirror is two times of the rotational angle. The laser beam is positioned on the mirror shown in Figure 3(e), and then we measure the optical reflection angle of the proposed 2-D scanning mirror system. A screen covered with graph paper is located 200mm away from the mirror and the laser beam reflected from the mirror generates various scanning profiles.

### 4.2. Experimental Results

**Figure 4.**
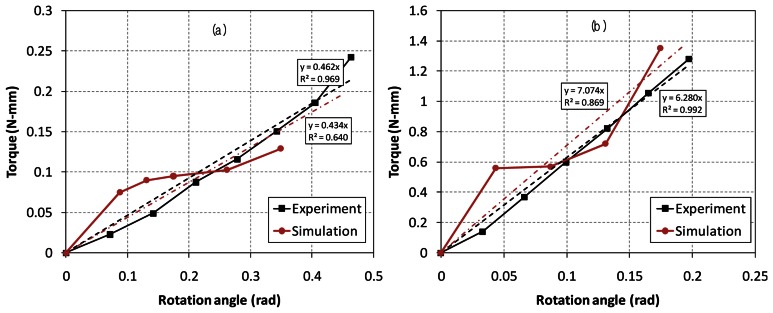
Experiment and simulation results of restoring constants. (**a**) X-axis, (**b**) Y-axis.

**Figure 5.**
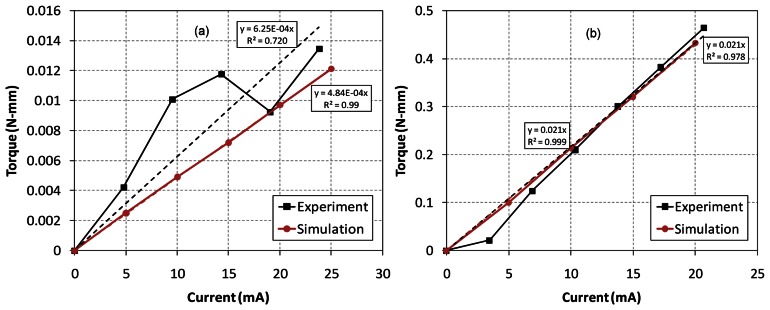
Experiment and simulation results of torque constants. (**a**) X-axis, (**b**) Y-axis.

The restoring and torque constants *K_m_* and *K_t_* at each axis are measured and the experimental results are compared with the FEA simulations in Figures 4 and 5, respectively. In order to obtain the restoring and torque constants at each axis, we measure torques about two axes as a function of the rotational angle and input current. Firstly, magnetic forces are measured using the load cell for the cases of different rotational angles with zero current at each coil. Here, the torque becomes the measured force multiplied by the distance from the axis to the measured point of mirror. Then the restoring constant *K_m_* is calculated from the linear Equation (2), which is the slope of the torque-angle curve in Figure 4.

Similarly, the torque constant *K_t_* is also calculated from the electromagnetic forces measured using the load cell for the cases of different input currents while the mirror and the whole frame are hold without moving. The torque constant is then the slope of the torque-current curve in Figure 5. We repeat the same experiments and calculate the average values of the constants using a least square method. In the Figures 4 and 5, the dashed lines are the linearized curves for the experiment and simulation results with the solid lines. The slopes of the linearized curves indicate the restoring and torque constants based on the linear models of Equations (2) and (3). Although the electromagnetic simulations and experiments are inherently nonlinear and very sensitive to the design parameters, most of the coefficients of determination (*R*^2^) are relatively close to 1. The linearized dynamic model is said to be valid in the electromagnetic simulations and experiments. The physical parameters of the two electromagnetic actuators are summarized in Table 1.

**Figure 6.**
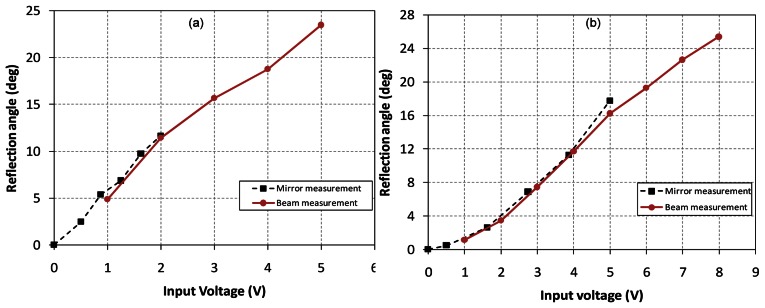
Static responses to input voltage. (**a**) reflection angle at X-axis, (**b**) reflection angle at Y-axis.

Then, we measure and calculate the static response such as the rotational angle as a function of the input voltage using the laser displacement sensor (Keyence^tm^ LB 081) and a potable laser beam. In the case of the moving-coil-type actuator around X-axis, the optical reflection angle is 15.7 degrees at the input voltage of ±3 V (±70 mA). In the case of the Y-axis actuator acting as the moving-magnet type, the optical reflection angle is 16.2 degrees at the input voltage of ±5 V (±34 mA). Experimental reflection angles measured at the mirror and beam spots are shown in Figure 6. The experimental results show that the dependence of the rotational angle on the input voltage is approximated to be linear. Therefore, the proposed 2-D actuators can be utilized as the mirror-rotating mechanism of a laser pointing device.

**Figure 7.**
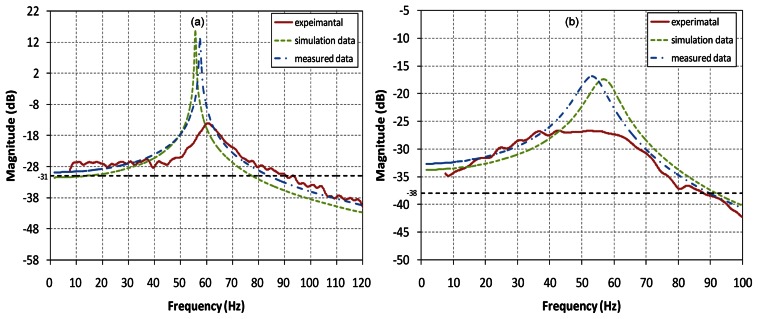
Frequency response plots of the proposed scanning mirror actuator. (**a**) X-axis, (**b**) Y-axis.

Furthermore, we performed experiments to measure the dynamic performances of the actuator system using the sinusoidal input. The experimental frequency response of the scanning mirror is plotted in Figure 7. It is also compared with the theoretical frequency responses by the Equation (5) for two cases using simulation and experimental data of the restoring and torque constants *K_m_* and *K_t_*. The upper moving-coil type actuator along X-axis has the resonance frequency of 60 Hz with the input voltage of ±3 V (±70 mA) which is the peak value in Figure 7(a). The optical reflection angle at the resonance frequency is about 90 degrees. In control systems, the bandwidth is defined as the frequency at which the frequency response has declined 3 dB (0.708 ratio) from its low-frequency value. Therefore, the frequency response plot in Figure 7(a) shows that the corresponding bandwidth is calculated to be about 91 Hz where the magnitude has a lower 3 dB than −28 dB at the low-frequency region. Similarly, the lower moving-magnet type actuator around Y-axis has the optical reflection angle of 50 degrees at the resonance frequency of 60 Hz when the applied voltage is ±5 V (±34 mA). It has the bandwidth of 88 Hz where the frequency response plot crosses the line of the magnitude−38 dB subtracting 3dB from the low frequency value (−35 dB) in Figure 7(b).

**Figure 8.**
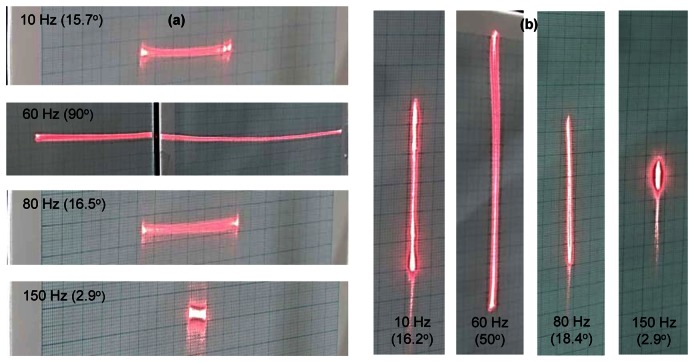
Laser scan patterns for various driving frequencies with the input voltage of (**a**) ±3 V at X-axis, (**b**)±5 Vat Y-axis.

Laser beam profiles by the proposed scanning mirror about X and Y axes are shown in Figure 8(a,b), respectively. The line scanning patterns at each axis are measured for various driving frequencies with the input voltage of ±3 V at X-axis and ±5 V at Y-axis. The line scan patterns at both axes have maximum angles at the driving frequency of 60 Hz, which is close to the resonance frequencies of both actuators. The scanning motion of the mirror system is affected by the fabrication error, the assembly clearance at each axis, the roughness of the mirror and the curvature of the projection surface.

**Figure 9.**
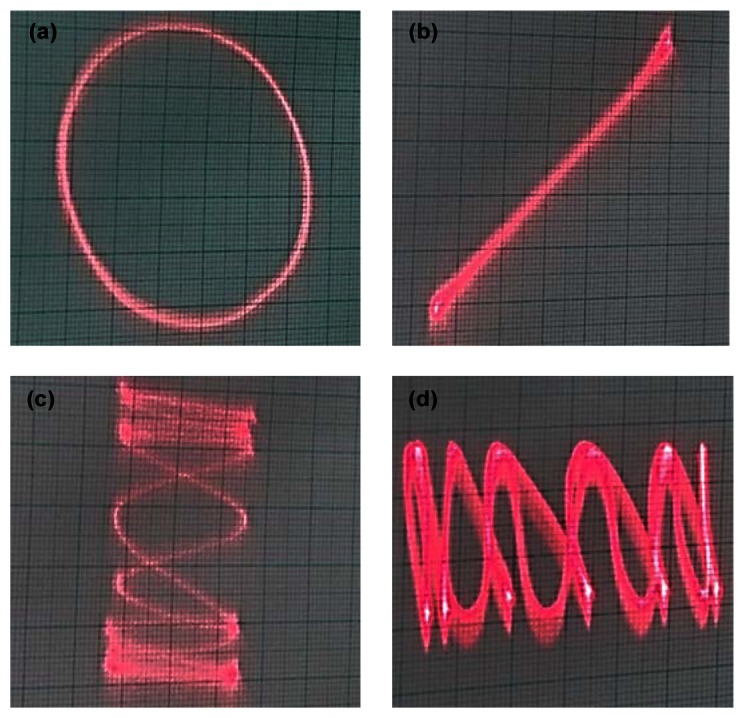
Laser beam profiles by the 2-D scanning mirror actuator with input voltages of ±3 V at X-axis and ±5 V at Y-axis. (**a**) circle profile by the driving frequency of 80 Hz at each axis, (**b**) line profile by 80 Hz at each axis, (**c**) 100 Hz at X-axis and 10 Hz at Y-axis, (**d**) 10 Hz at X-axis and 100 Hz at Y-axis.

Figure 9(a,b) shows the 2-D scanning profiles of laser beam when both actuators are driven by input voltages of ±3 V at X-axis and ±5 V at Y-axis. They show the circle and line patterns of laser beam when the same driving frequencies are applied. There is no hysteretic motion in the line pattern, indicating that the motion-decoupling actuators cause independent 2-D motions of the mirror scanning system. Figure 9(c,d) indicates that the scanning mirror actuator can follow various driving patterns.

Since the proposed scanning mirror actuator is larger than those of MEMS or micromachining based mirror actuators, the resonance frequencies of the two actuators are lower than those of the electromagnetically actuated micro mirrors. However the optical reflection angles of the proposed actuator are not only similar or larger than those of the MEMS based mirror actuators, but the dynamic range of the proposed 2-D mirror actuator is also even larger. The dynamic bandwidth of the actuator meets the design specifications for industrial applications such as imaging devices like confocal laser scanning microscopes, optical coherence topographies, scanning laser rangefinder, projectors and laser marking machines.

## 5. Conclusions

We propose a novel 2-D scanning mirror system using electromagnetic actuators with a simple structure composed of one magnet and two coils. The mirror-actuating device combines two electromagnetic actuators of the conventional moving-coil and the moving-magnet types, generating 2-D scanning motions; the rotation of the mirror part around X-axis and the rotation of the whole frame around Y-axis. From FEA simulations and experiments, it is proved that the proposed system has good static performance and large reflection angle, moderate bandwidth and broad dynamic range at both orthogonal directions. The proposed 2-D mirror system is suitable for the commercial and industrial applications of various laser scanning devices.

## Figures and Tables

**Table 1.** Physical parameters of 2-D scanning mirror actuators.

	***J* Inertia (Kg/m^2^)**	***R* Resister (Ohm)**	***K_t_* Torque constant (Nm/A)**		***K_m_* Restoring constant (Nm/rad)**	
	
			**Simulation**	**Experiment**	**Simulation**	**Experiment**
X-axis	3.58e-9	42	4.84e-4	6.25e-4	4.34e-4	4.62e-4
Y-axis	55.65e-9	145	2.14e-2	2.17e-2	7.07e-3	6.28e-3
